# Evaluations of Antioxidant Enzyme Activities, Total Sialic Acid and Trace Element Levels in Coronary Artery Bypass Grafting Patients

**DOI:** 10.21470/1678-9741-2020-0162

**Published:** 2021

**Authors:** Damla Yildiz, Suat Ekin, Sahin Sahinalp

**Affiliations:** 1 Department of Chemistry, Division of Biochemistry, Faculty of Science, Van Yuzuncu Yil University, Van, Turkey.; 2 Department of Cardiovascular Surgery, Faculty of Medicine, Van Yuzuncu Yil University, Van, Turkey.

**Keywords:** Coronary Artery Disease, Trace Elements, Glutathione Peroxidase, Superoxide Dismutase, Catalase, Total Sialic Acid, Malondialdehyde

## Abstract

**Introduction:**

In this study, patients before and after cardiac surgery with cardiopulmonary bypass (CPB) and control subjects were evaluated for erythrocyte glutathione peroxidase, catalase and superoxide dismutase enzyme activities, in addition to glutathione, malondialdehyde, serum total sialic acid, lipid-bound sialic acid, total antioxidant status, trace elements and mineral levels. The correlation of these variables with coronary artery disease (CAD) was also assessed.

**Methods:**

A total of 30 CAD patients and 30 control subjects were included in the study. CAD patients were divided into three groups: before surgery (BS), first day after surgery (1^st^ day AS) and seventh day after surgery (7^th^ day AS).

**Results:**

Malondialdehyde (MDA) and total sialic acid (TSA) levels were significantly higher in CAD (BS) than in the control group (*P*<0.05, *P*<0.05). In addition, GSH and TAS levels were significantly lower in the 1st day AS group than in the control group (*P*<0.001, *P*<0.01). Moreover, Co, Cu, Mg, Se, V and Zn levels were significantly lower in CAD (BS) group than in the control group (*P*<0.01, *P*<0.01, *P*<0.01, *P*<0.01, *P*<0.05, *P*<0.001).

**Conclusions:**

It was concluded that the levels of LDL-C, total cholesterol, triglycerides and CRP significantly associated with parameters, as well as Cu, Ca and SOD activity, should be measured together to monitor CAD. It is also considered that measuring TSA and MDA might be an appropriate choice for biomarkers of CAD.

**Table:** 

Abbreviations, acronyms & symbols				
**ABTS**	**= 2,2-azinobis-3-ethylbenzothiazoline-6-sulfonate**		**GSH-Px**	**= Glutathione peroxidase**
**ANOVA**	**= Analysis of variance**		**LDL-C**	**= Low-density lipoprotein cholesterol**
**BMI**	**= Body mass index**		**LSA**	**= Lipid bound sialic acid**
**BS**	**= Before surgery**		**MDA**	**= Malondialdehyde**
**CA**	**= Calcium**		**OSI**	**= Oxidative stress index**
**CABG**	**= Coronary artery bypass grafting**		**ROS**	**= Reactive oxygen species**
**CAD**	**= Coronary artery disease**		**SOD**	**= Superoxide dismutase**
**CAT**	**= Catalase**		**TAS**	**= Total antioxidant status**
**CPB**	**= Cardiopulmonary bypass**		**TE**	**= Trace elements**
**CRP**	**= C-reactive protein**		**TOS**	**= Total oxidant status**
**Cu**	**= Copper**		**TSA**	**= Total sialic acid**
**GSH**	**= Reduced glutathione**			

## INTRODUCTION

Coronary artery disease (CAD), which is mediated by multiple interactions of environmental and genetic factors, is the main cause of disability and death worldwide ^[1]^. Oxidative stress and chronic inflammation may play a role in the pathogenesis of CAD and atherosclerosis ^[2]^. The risk factors due to excess free radicals are smoking, diet, pollution and metabolic abnormalities that lead to increased oxidative stress to the heart ^[3]^. A series of inflammatory and immunological alterations, which trigger oxidative stress, may be involved during cardiac surgery with cardiopulmonary bypass (CPB) ^[4]^.

Reactive oxygen species (ROS) upregulate atherosclerotic events such as cell infiltration, platelet activation, adhesion and migration. These ROS oxidize cellular biomolecules, including lipids, nucleic acids and proteins, causing endothelial impairments ^[5]^. The unbalanced production of ROS could trigger protein and lipid oxidation in the vascular wall ^[2,6]^. One of the main risk factors for CAD is hyperlipidaemia. The incidence of CAD is higher in the group of patients with low-density lipoprotein cholesterol values ^[7]^. Destruction of endothelium, disruption of the routine vasomotor system and increase in thrombosis thought to be caused by ROS are the mechanisms of atherogenesis ^[8]^.

In energy transfer and physiological heart function, micronutrients, which are enough in trace amount, are fundamental cofactors. Micronutrients are compounds that inhibit oxidation and play a role in signal transduction pathways ^[9]^. Trace elements (TE) play an important role in the balance of normal structure and physiological functions of cells. The cardiovascular system may be directly affected by TE levels and may also be affected indirectly in the vascular system through the role of trace elements in lipid metabolism ^[10]^. Mg is essential, hence, it participates in many biochemical, physiological and cellular processes for an appropriate cardiovascular function ^[11]^.

The purpose of this study was to evaluate the alterations in erythrocytes glutathione peroxidase (GSH-Px), catalase (CAT), superoxide dismutase (SOD) enzyme activities, the glutathione (GSH) and malondialdehyde (MDA) levels, in the serum total sialic acid (TSA), lipid bound sialic acid (LSA), total antioxidant status (TAS), total oxidant status (TOS), oxidative stress index (OSI), trace element (As, Be, Cu, Cr, Co, Pb, Li, Sr, Se, Ti, Zn, V), mineral (Mg, Ca, Na, Cl, K), lipid profile parameters (total cholesterol, LDL-C, triglycerides) and some biochemical parameters (albumin, ALT, AST, BUN, CRP, glucose, uric acid, Ca, K, Na, Cl) in relation to indices of CAD in patients before (BS) and in the first and seventh day after surgery (1^st^ day AS and 7^th^ day AS) with the use of CPB to compare the control subjects. In addition, the correlations between the parameters were used to evaluate the relationship with CAD.

## METHODS

### Study Population

Before the beginning of the study, the necessary approval was received from the ethics committee of the Faculty of Medicine of the Van Yuzuncu Yil University (Van YYU) (Van YYU, 28.02.2017/Decision no. 2017/09). The study was conducted in accordance with the principles of the Declaration of Helsinki. All patients were properly informed about the study and signed a written approval form. Patients with coronary artery disease were examined at the Department of Cardiovascular Surgery at Van YYU.

A total of 30 CAD patients and 30 healthy subjects were included in the study. CAD patients were divided into before surgery (BS), after coronary artery bypass grafting (CABG) with CPB (1^st^ day AS) and after CABG with CPB (7^th^ day AS) groups. The subjects met the inclusion criteria for a wide range of etiology and CAD manifestations. The variables collected from each patient were: sex, age, weight and height.

All patients underwent coronary angiography to assess the severity of CAD at the Faculty of Medicine of the Van Yuzuncu Yil University. CAD was identified according to the guidelines of the European Association for Cardio-Thoracic Surgery and the European Society of Cardiology on the practice of myocardial revascularization ^[12]^. CAD was defined as significant left main coronary stenosis >50%, proximal left anterior descending stenosis >50%, presence of multivessel stenosis (narrowing >50% in coronary arteries) and complex coronary lesions. CAD patients were selected from candidates for CPB.

All CAD patients were operated using a conventional CPB circuit (C5, Sorin Group, Germany). Cardiac surgeries with CPB were conducted under moderate hypothermia (28-32 °C). Instantly after the cross-clamping, cardiac arrest was caused by an antegrade infusion of cold blood cardioplegic solution. The CPB circuit was prefilled with 1500 mL of crystalloid priming solution, 250 mL of mannitol and 0.1 mL of 8.4% sodium bicarbonate.

Patients were screened out according to major exclusion criteria, including previous coronary surgery, infectious diseases, malignancy, any history of surgery and other known chronic diseases (liver and renal failure etc.). Healthy subjects were selected from the healthy population with normal electrocardiographic findings. This group had a normal lipid profile, with no history of cardiovascular disease and known chronic diseases. Participant information was obtained by questionnaires on personal data and clinical measurements such as age, gender and drug use during the past months.

### Sample Preparation and Analytical Methods

The first blood sample was taken from each patient for study after 12 hours of fasting in the before surgery (BS) group. The second sample was taken within the first 24 hours after patients were submitted to CABG with CPB (1^st^ day AS group). The third blood sample was taken on the day of discharge after CABG with CPB (7^th^ day AS group). Seven mL of venous blood were obtained after 12 hours of fasting and the blood was centrifuged for 10 minutes at 2550 rpm at 4 °C. The serum was separated and kept in covered tubes at -65 °C, until the determination of TAS, TOS, TSA, LSA, mineral and trace element.

Erythrocytes were obtained as whole blood samples from fasting subjects by venous puncture with heparinized vials. Blood was centrifuged at 1000 × g and the plasma was removed by aspiration. Erythrocytes were washed three times with ice-cold saline (0.153 mol/L NaCl) and centrifuged at 1000 rpm for 5 minutes. The erythrocytes were then processed shortly for measurement of MDA and GSH. The erythrocytes were stored at -65°C until the assay of SOD, CAT, and GSH-Px enzyme activities.

Determinations of serum concentrations of TE (arsenic [As], beryllium [Be], copper [Cu], chromium [Cr], cobalt [Co], lead [Pb], lithium [Li], strontium [Sr], selenium [Se], titanium [Ti], Zinc [Zn], vanadium [V], and mineral (magnesium (Mg) were carried out by ICP-OES, with a Thermo ICP-OES iCAP 6300 DUO device (Thermo Fisher Scientific Inc, UK). Multi-element reference materials (Inorganic ventures IV-Stock-8) were used.

Measurements of biochemical parameters: albumin, aspartate transaminase (AST), blood urea nitrogen (BUN), alanine transaminase (ALT), C-reactive protein (CRP), glucose, triglyceride (TG), total cholesterol (TC), low-density lipoprotein cholesterol (LDL-C), uric acid (UA), sodium (Na), calcium (Ca), potassium (K) and chloride (Cl) were carried out using standard methods with the biochemical analyzer Architect CI16200 (Abbott Diagnostics, Abbott Park, IL) at the laboratory of the Faculty of Medicine of Van YYU. The level of CRP was measured by a nephelometer (Siemens BN II, Siemens Healthcare GmbH).

Erythrocyte GSH was analysed according to the method described by Rizzi et al. ^[13]^. Erythrocyte MDA was evaluated using the method defined by Jain et al. ^[14]^ Erythrocyte activities of antioxidant enzymes (GSH-Px, CAT and SOD) were evaluated by a spectrophotometer (Shimadzu UV-1800 UV-VIS, Kyoto). The results are expressed as enzyme units per gram of haemoglobin (IU/g Hb). Erythrocyte GSH-Px (E.C.1.11.1.9) activity was evaluated at a wavelength of 340 nm according to the method described by Paglia et al. ^[15]^. SOD (EC 1.15.1.1) activity was measured at a wavelength of 505 nm based on the method described by Sun et al. ^[16]^. CAT (EC 1.11.1.6) activity was measured at a wavelength of 240 nm by the decomposition of H_2_O_2_, using the method described by Aebi ^[17]^. Serum TSA level was determined according to the method described by Sydow et al. ^[18]^. Serum LSA level was determined using the method specified by Katopodis et al. ^[19]^.

The total antioxidant status (TAS) is often used to evaluate the overall antioxidant state of the body and to monitor antioxidant therapy. The serum TAS was determined with 2,2-azinobis-3-ethylbenzothiazoline-6-sulfonate (ABTS) and was measured at a wavelength of 660 nm. The results are expressed as mmol Trolox equivalent/L ^[20]^. The total oxidant status (TOS) was used to determine the patient’s overall oxidative status. Serum total oxidation status (TOS) was measured at 530 nm using method, developed by Erel. The results are expressed in terms of millimoles of hydrogen peroxide (H_2_O_2_) equivalent per liter (mmol H_2_O_2_ equivalent/L) ^[21]^.

The oxidative stress index (OSI) is defined as the ratio between the TOS and TAS levels. The OSI is an indicator of the degree of oxidative stress. The OSI was calculated using the ratio between the TOS and TAS levels.

$\mathrm{OSI}=\frac{\mathrm{TOS}\;\left(µ\mathrm{mol}\;H2O2\;\mathrm{Eq}\mathrm{./}L\right)}{\mathrm{TAS}\;\left(\mathrm{mmol}\;\mathrm{trolox}\;\mathrm{Eq}\mathrm{./}L\right)}X1\mathrm{00}$

### Statistical Analysis

The results are expressed as mean±standard error of the mean (±SEM). The analysis of variance (ANOVA) or the Kruskal-Wallis test was used for statistical analysis, and then Tukey test was carried out for *post-hoc* comparison of mean values. Pearson's correlation analysis was used to assess the levels of biochemical parameters for correlation studies in the coronary artery disease (CAD) in patients of the BS group. The statistical analysis was performed using the statistical software SPSS® v23 (SPSS Inc., Chicago, Il, USA).

## RESULTS

The comparison of clinical and biochemical characteristics in CAD patients and control subjects is shown in Table 1.

**Table 1:** Distribution of clinical and biochemical characteristics of CAD patients and control subjects.

Parameters	Control	CAD patients
	(®X±SEM)	(®X±SEM)
Gender (M/F)	20/10	18/12
Age (years)	62.97±1.84	63.23±1.79
BMI (kg/m^2^)	25.48±0.72	25.95±0.60
CPB time (min)	-	86.42±7.57
Duration of surgery (min)	-	207±6.60
Ischemia time (min)	-	62.38±6.54
Albumin (g/dL)	4.07±0.06^b^	3.59±0.15^b^
ALT (U/L)	21.71±2.47	22.11±3.57
AST (U/L)	26.56±2.59	33.06±5.53
BUN (mg/dL)	32.80±1.92^c^	44.92±4.44^c^
CRP (mg/L)	10.64±3.97^b^	31.74±6.53^b^
Glucose (mg/dL)	97.16±2.29^b^	137±11.40^b^
Total cholesterol (mg/dL)	168.90±6.40^c^	195.40±12.06^c^
LDL (mg/dL)	103.20±6.63^b^	134.90±9.15^b^
Triglycerides (mg/dL)	118.00±9.71^c^	182.1188±34.84^c^
Uric acid (mg/dL)	4.94±0.20	5.83±0.59
Ca (mmol/L)	2.28±0.04^b^	2.13±0.02^b^
Na (mmol/L)	139.20±0.40	138.80±0.52
Cl (mmol/L)	108.10±0.59^a^	103.30±0.98^a^
K (mmol/L)	4.40±0.07	4.28±0.09
HCT (%)	43.08±1.08^c^	39.77±1.11^c^
HGB (g/dL)	14.01±0.35	13.35±0.36
WBC (10^3 ^mL)	7.52±0.49^b^	9.55±0.57^b^
PLT (10^3^ mL)	300.60±22.44^b^	218.60±13.46^b^

a *P* <0.001,

b *P* <0.01,

c *P* <0.05 (different letters, significant differences between groups).

^a,b,c^ Significant difference by one-way analysis of variance (ANOVA).

As shown in Table 1, the age of healthy controls and CAD patients was 62.97±1.84 and 63.23±1.79 years and the body mass index (BMI) of the healthy group and CAD patients was 25.48±0.72 kg/m^2^ and 25.95±0.60 kg/m^2^. No significant difference was observed between CAD and control groups in terms of age and BMI (*P*>0.05).

According to the statistical analysis, comparison of CAD patients and controls indicated a significant increase in BUN (*P*<0.05), CRP (*P*<0.01), glucose (*P*<0.01), total cholesterol (*P*<0.05), TG (*P*<0.05), LDL (*P*<0.01), WBC (*P*<0.01) and a significant decrease in albumin (*P*<0.01), Ca (*P*<0.01), Cl (*P*<0.001), HCT (*P*<0.05), PLT (*P*<0.01) concentrations. However, a comparison of ALT, AST, uric acid, Na, K, and HGB levels revealed no statistical significance between groups of CAD patients and controls (*P*>0.05).

Table 2 lists CAT, SOD, GSH-Px enzyme activities, in addition to GSH, MDA, LSA, TSA, TAS, TOS and OSI levels in CAD, before and after CABG with CPB and in controls.

**Table 2:** Mean levels of antioxidant enzymes (SOD, GSH-Px, CAT activities), MDA, GSH, TSA, LSA, TAS, TOS and OSI in CAD patients, before and after surgery (AS1st day, AS7th day) with CPB and in controls.

Parameters	Control	CAD (BS)	CAD (1^st ^day AS)	CAD (7^th ^day AS)
	(®X±SEM)	(®X±SEM)	(®X±SEM)	(®X±SEM)
GSH-Px (IU/g Hb)	27.71±1.52^b^	21.44±1.26^b^	23.12±1.29	24.93±1.34
CAT (IU/g Hb)	1185.16±37.74^b,c,c1^	1015.17±31.21^b^	1027.46±42.66^c^	1017.26±35.73^c1^
SOD (IU/g Hb)	1873.83±36.53	1819.46±34.57	1769.65±29.71	1825.45±34.78
GSH (µmol/g Hb)	1.09±0.043^a,a1,c^	0.79±0.026^a,a2^	0.80±0.027^a1,b^	0.97±0.028^a2,b,c^
MDA (nmol/g Hb)	9.35±0.54^a,b,c^	14.06±1.49^c^	15.63±1.12^a^	15.17±1.07^b^
TSA (mmol/L)	1.50±0.075^b,c,c1^	1.92±0.103^c^	2.01±0.105^b^	1.89±0.099^c1^
LSA (mmol/L)	0.285±0.0020^a^	0.292±0.0023	0.297±0.0025^a,b^	0.287±0.0018^b^
TAS (mmol Trolox Eq/L)	3.20±0.057^b,b1,b2^	2.89±0.063^b^	2.85±0.053^b1^	2.88±0.088^b2^
TOS (µmolH_2_O_2_Eq/L)	22.30±1.153^b^	26.08±1.147	27.92±1.087^b^	26.62±1.288
OSI	0.71±0.0405^a,b,c^	0.91±0.0425^c^	0.99±0.0432^a^	0.95±0.0557^b^

^a,a1,a2^*P* <0.001, b,b1,b2: *P* <0.01, ^c,c1^*P* <0.05 (different letters, significant differences between groups).

^a,a1,a2, b,b1,b2, c,c1 ^Significant difference by one-way analysis of variance (ANOVA).

**Fig. 1:**
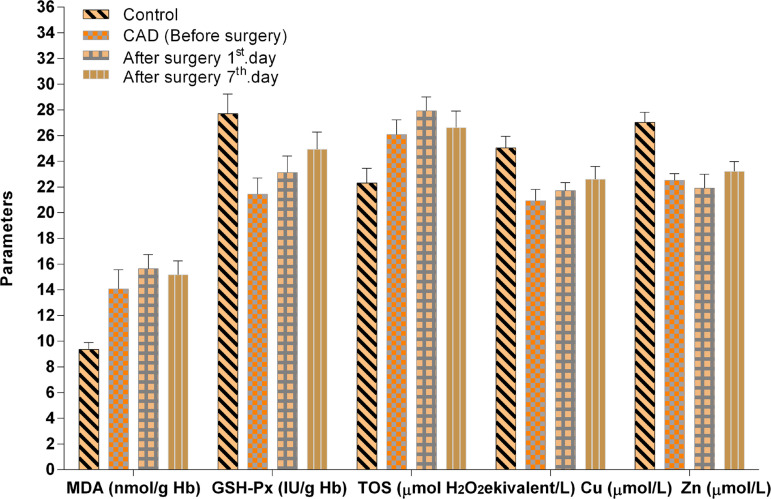
MDA, GSH-Px, TOS, Cu and Zn levels in CAD patients, before and after surgery (AS1st day, AS7th day) and in control groups. Values represent mean±standard error of the mean (XSEM).

**Fig. 2:**
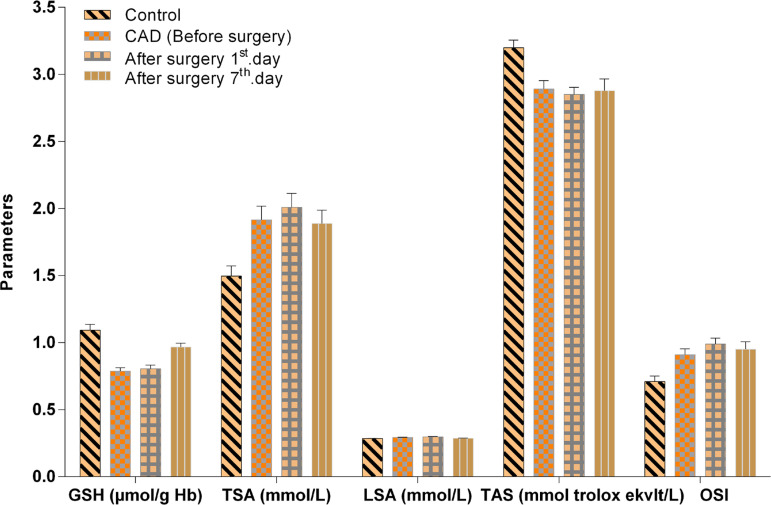
GSH, TSA, LSA, TAS and OSI levels in CAD patients, before and after surgery (AS1st day, AS7th day) and in control groups. Values represent mean±standard error of the mean (XSEM).

**Fig. 3:**
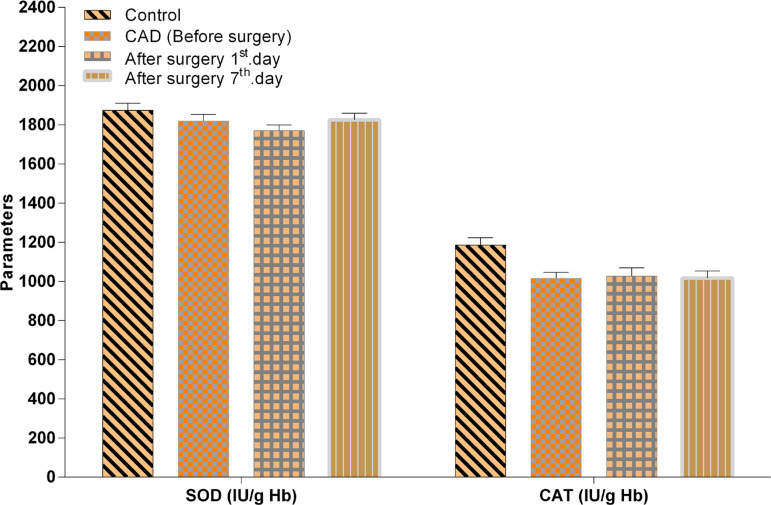
SOD and CAT enzyme activities in CAD patients, before and after surgery (AS1st day, AS7th day) and in control groups. Values represent mean±standard error of the mean (XSEM).

It was observed that a significant reduction in GSH-Px and CAT enzyme activities, GSH, TAS (*P*<0.01, *P*<0.01, *P*<0.001, *P*<0.01), and MDA, TSA and OSI concentrations (*P*<0.05, *P*<0.05, *P*<0.01) were significantly higher in the CAD (BS) group than in the healthy control group. However, the after surgery with CPB (1^st^ day AS) group was also significantly lower than the control group regards to CAT, TAS and GSH (*P*<0.05, *P*<0.01, *P*<0.001, respectively), and a significant increase in MDA, TSA, LSA, TOS, OSI concentrations (*P*<0.001, *P*<0.01, *P*<0.001, *P*<0.01, *P*<0.001). CAT enzyme activity, GSH and TAS (*P*<0.05, *P*<0.05, *P*<0.01) were significantly lower than the control group in the after surgery with CPB (7^th^ day AS) group, while the GSH level was lower in the CAD (BS) group than after surgery with CPB (7^th^ day AS) (*P*<0.001) group. Similarly, the GSH level was significantly lower and the LSA level was importantly higher in the after surgery with CPB (1^st^ day AS) group than in the after surgery with CPB (7^th^ day AS) group (*P*<0.01, *P*<0.01). However, the comparison of the SOD enzyme activity revealed no statistical significance between groups of CAD patients and healthy controls (*P*>0.05) (Figures 1 to 3).

The mean trace element (As, Be, Cu, Cr, Co, Pb, Li, Sr, Se, Ti, Zn, V), mineral (Mg) and trace element ratio (Cu/V, Se/Co, V/Zn) levels of CAD patients before, after undergoing CABG with CPB and control subjects are shown in Table 3.

**Table 3:** Mean trace element (As, Be, Co, Cr, Cu, Li, Pb, Se, Sr, Ti, V, Zn), mineral (Mg) and trace element ratio (Cu/V, Se/Co, V/Zn) levels in CAD patients, before and after surgery (1st day AS, 7th day AS) with CPB and in controls.

Parameter	Control	CAD (BS)	CAD (1^st^ day AS)	CAD (7^th^ day AS)
(µmol/L)	®X±SEM	®X±SEM	®X±SEM	®X±SEM
As (µmol/L)	0.663±0.12	0.694±0.13	0.686±0.14	0.677±0.15
Be (µmol/L)	0.491±0.040	0.466±0.073	0.489±0.055	0.487±0.039
Co (µmol/L)	0.205±0.0083^b,b1,c^	0.171±0.0082^b^	0.169±0.0077^b1^	0.174±0.0053^c^
Cr (µmol/L)	0.51±0.07	0.44±0.09	0.42±0.06	0.45±0.10
Cu (µmol/L)	25.04±0.91^b,c^	20.93±0.88^b^	21.72±0.62^c^	22.61±0.98
Li (µmol/L)	3.21±0.47	3.43±0.48	3.31±0.56	3.29±0.57
Pb (µmol/L)	0.506±0.0253	0.521±0.0411	0.519±0.0287	0.514±0.0246
Se (µmol/L)	1.64±0.058^b,b1,c^	1.36±0.056^b^	1.35±0.046^b1^	1.38±0.070^c^
Sr (µmol/L)	1.821±0.08	1.866±0.10	1.818±0.15	1.896±0.13
Ti (µmol/L)	0.695±0.0829	0.720±0.0921	0.739±0.0695	0.719±0.0827
V (µmol/L)	6.50±0.133^c,c1,c2^	5.91±0.156^c^	5.96±0.130^c1^	5.94±0.100^c2^
Zn (µmol/L)	27.02±0.79^a,a1,b^	22.51±0.53^a^	21.91±1.08^a1^	23.21±0.77^b^
Mg (mmol/L)	1.10±0.0262^b,c^	0.95±0.0345^b^	0.96±0.0339^c^	1.02±0.0343
Cu/V	3.91±0.16	3.49±0.22	3.70±0.14	3.85±0.18
Se/Co	8.334±0.483	7.906±0.345	8.325±0.456	8.215±0.578
V/Zn	0.2477±0.0100^c^	0.269±0.0108	0.2901±0.0144^c^	0.2637±0.0093

^a,a1^*P* <0.001, ^b,b1^*P* <0.01, ^c,c1,c2^*P* <0.05 (different letters, significant differences between groups).

^a,a1, b,b1, c,c1,c2^ Significant difference by one-way analysis of variance (ANOVA).

**Fig. 4:**
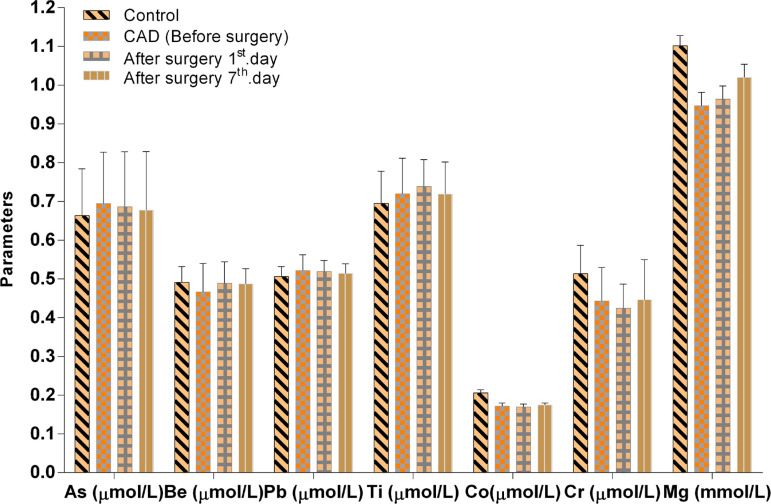
As, Be, Pb, Ti, Co, Cr and Mg levels in CAD patients, before and after surgery (AS1st day, AS7th day) and in control groups. Values represent mean±standard error of the mean (XSEM).

**Fig. 5:**
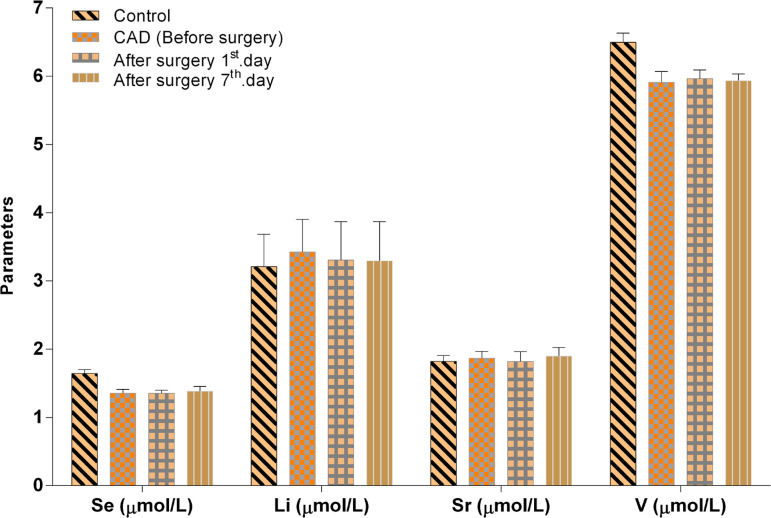
Se, Li, Sr and V levels in CAD patients, before and after surgery (AS1st day, AS7th day) and in control groups. Values represent mean±standard error of the mean (XSEM).

The Co, Cu, V, Se, Zn and Mg levels in the CAD (BS) group were significantly lower than in the healthy control group (*P*<0.01, *P*<0.01, *P*<0.05, *P*<0.01, *P*<0.001, *P*<0.01). Moreover, the after surgery with CPB (1^st^ day AS) and control groups demonstrated important differences in the concentrations of Co, Cu, Se, V, Zn, Mg and V/Zn ratio (*P*<0.01, *P*<0.05, *P*<0.01, *P*<0.05, *P*<0.001, *P*<0.05, *P*<0.05); data also showed important increase in the V/Zn (*P*<0.05) ratio. Whereas, the difference between the after surgery with CPB (7^th^ day AS) and control groups in Co, Se, V and Zn concentrations decreased significantly (*P*<0.05, *P*<0.05, *P*<0.05, *P*<0.01). However, a comparison of the As, Be, Cr, Li, Pb, Sr, Ti, Cu/V and Se/Co levels revealed no statistical significance between CAD patients and controls (*P*>0.05) (Figures 4 and 5).

Table 4 demonstrates significant correlations between the variables of the CAD (BS) patients’ group. Associations between Se, Co, Zn, Cu, K, Mg, Ca, Cl, SOD activity, GSH, TAS, LSA, ALT, AST, LDL, total cholesterol, glucose, CRP, albumin, triglyceride, BMI and age in CAD patients (BS) group were calculated with Pearson’s correlation coefficient r and significance level *P*.

**Table 4:** Pearson correlations between the parameters in CAD patients (before surgery).

Parameters	r	*P*
Se-Co	0.621	0.001
Se-Mg	0.412	0.033
K-Co	-0.568	0.001
K-GSH	0.421	0.020
K-ALT	-0.405	0.029
K-AST	-0.489	0.040
LDL-C-total cholesterol	0.639	0.008
Glucose-Ca	-0.529	0.011
GSH-Co	-0.460	0.012
BMI-GSH	0.460	0.031
GSH-Se	-0.414	0.032
BMI-Cl	-0.537	0.012
Triglycerides-Ca	-0.672	0.023
SOD-Zn	0.399	0.029
AST-LSA	0.510	0.031
Age-TAS	0.381	0.038
Age-K	0.424	0.020
CRP-albumin	-0.533	0.041
Total cholesterol-Cu	-0.511	0.043
Cl-Mg	0.376	0.049
ALT-TAS	-0.368	0.049

To assess the correlation analysis in CAD (BS) cases, a positive linear correlation was found between the levels of Se, Co and Mg in subjects with CAD (BS) (r=0.621; *P*=0.001, r=0.412; *P*=0.033), between K and GSH, age levels (r=0.421; *P*=0.020, r=0.424; *P*=0.020), LDL-total cholesterol (r=0.639; *P*=0.008), BMI-GSH (r=0.460; *P*=0.031), SOD-Zn (r=0.399; *P*=0.029), AST-LSA (r=0.510; *P*=0.031), age-TAS (r=0.381; *P*=0.038) and Cl-Mg (r=0.376; *P*=0.049). In addition, significantly negatively correlated between the concentration of K and Co, ALT, AST levels (r=-0.568; *P*=0.001, r=-0.405; *P*=0.029, r=-0.489; *P*=0.040), between GSH and Co, Se levels (r=-0.460; *P*=0.012, r=-0.414; *P*=0.032), glucose-Ca (r=-0.529; *P*=0.011), BMI-Cl (r=-0.537; *P*=0.012), TG-Ca (r=-0.672; *P*=0.023), CRP-albumin (r=-0.533; *P*=0.041) and total cholesterol-Cu (r=-0.511; *P*=0.043) in CAD (BS) patients.

## DISCUSSION

In this study, in CAD patients before and after CABG with CPB and in healthy control groups, erythrocytes antioxidant enzyme activities (GSH-Px, CAT and SOD), GSH, MDA in serum TAS, TOS, TSA, LSA, TE (As, Be, Cu, Cr, Co, Pb, Li, Sr, Se, Ti, Zn, V), mineral (Mg) and biochemical parameters (total cholesterol, LDL-C, TG albumin, ALT, AST, BUN, CRP, glucose, uric acid, Ca, K, Na, Cl) levels were assessed, in addition, correlations between the parameters were to evaluate the relationship with CAD patients (before surgery).

CABG is a recognized therapeutic approach in people with symptomatic multivessel CAD. Cardiac surgery with CPB leads to ischemia reperfusion-mediated oxidative stress. Inflammation and oxidative stress are associated with the pathogenesis of atherosclerosis and increase the risk of postoperative atrial fibrillation and graft failure ^[22]^ Trace elements are necessary for a wide range of primary cellular functions and are especially crucial for various enzymes involved in the generation and neutralization of ROS that are normally produced by the cell ^[23]^. Trace elements such as Cu and Co may contribute to myocardial dysfunction ^[4]^.

To our knowledge, there is no published information on some parameters such as TSA, LSA, As, Be, Co, Be, As, Cr, Li, Ti, V, V/Zn levels in patients with CAD before and after surgery with CPB and correlations between biochemical parameters in CAD patients before surgery. In the current study, statistical analysis clearly demonstrates that comparison of CAD patients and controls indicated a significant elevation of TC (*P*<0.05), LDLC (*P*<0.01) and triglycerides (*P*<0.05) concentrations. In this study, significant increases in triglycerides, total cholesterol and LDL-C values were consistent with the literature ^[8]^. Via oxidation of LDL-C and free radical formation, the oxidative stress contributed to CAD pathogenesis ^[8]^. We also observed increased lipid peroxidation and oxidation of LDL among our CAD patients.

In a study by Lee et al. ^[7]^, conducted in erythrocyte samples collected from CAD patients, activities of erythrocyte SOD (*P*=0.034), CAT (*P*=0.033), GSH-Px, (*P*=0.042) were reported to have significantly decreased activity compared to the healthy control groups. In a study by Palazhy et al. ^[24]^, on the samples taken from patients with CAD, erythrocyte GSH-Px and GSH were reported to be significantly (*P*=0.001) and (*P*=0.001) decreased when compared to healthy control groups. However, a significant increase in MDA level (*P*=0.001) was found. Almzaiel et al. ^[8]^ observed that, in serum samples taken from patients with ischemic heart disease, the levels of Zn, Cu, SOD and CAT were importantly decreased (*P*<0.05, *P*<0.05, *P*<0.05 and *P*<0.05) when compared with healthy control groups. Conversely, LDL-C and triglycerides levels increase significantly (*P*<0.01, *P*<0.01). In another study, Tani et al. reported that the serum MDA level increased significantly in CAD compared with control group (*P*<0.001), whereas the TAS value was significantly lower in patient group (*P*<0.001) ^[25]^.

In the study, the results of reduced CAT, GSH-Px activities and GSH levels in CAD were consistent with the results of other studies ^[7,8,24]^, while findings of elevated SOD activity in CAD patients were not supported by other researchers ^[7,24]^. The significant increase in MDA value was consistent with those reported by other authors ^[22,24]^. However, the decrease in the TAS level agree with the data of Tani et al. ^[25]^.

In a different study by Bayir, serum values of Zn, Se and Cu in male and female patients in acute coronary syndrome and control groups were reported to be significantly (*P*<0.001, *P*<0.05, *P*<0.001) decreased compared to healthy control groups ^[10]^. The levels of Cu, Se and Zn in the CAD (BS) group were importantly lower than in the healthy group (*P*<0.01, *P*<0.01, *P*<0.001). However, a comparison of the levels of As, Be, Cr, Li, Pb, Sr, Ti, Cu/V and Se/Co revealed no statistical significance between CAD patients and controls (*P*>0.05). Our findings (the significant decrease in Zn, Se, Cu values) were in parallel with the results of other studies ^[8,10]^. These results show that low levels of Cu, Zn and Se can present a risk for CAD. It has been reported that the majority of cardiac surgery patients presented a significant Se deficiency before surgery, which was increased by the intra-operative procedure ^[22]^. There was no study determining the levels of trace elements As, Be, Co, Be, As, Cr, Li, Ti and V.

It was thought that Cu could contribute to myocardial dysfunction in heart failure, and that Cu deficiency decreases the activity of cardiac cytochrome c oxidase ^[10]^. Two of the most significant micronutrients are Zn and Cu, which play a major role in the oxidant/antioxidant mechanism ^[9]^. Cu deficiency leads to an increase in total cholesterol and in LDL-cholesterol, increasing its atherogenicity. Low level of Cu elevates the risk of CAD ^[8]^.

In the study, it was observed that the levels of Mg in the CAD (BS) group were significantly lower than in the healthy group (*P*<0.01). Moreover, the after surgery with CPB (1^st^ day AS) and control groups differed significantly in the Mg concentration (*P*<0.05). Mg is essential to the etiopathology of several cardiovascular diseases, including atherosclerosis, CAD, hypertension, cardiac arrhythmias and congestive heart failure ^[11]^.

The findings of the present study provide evidence that the inflammation marker (CRP) is intimately linked with CAD. Our results suggested that high V/Zn ratio, MDA, TSA, LSA, TOS, OSI and low Co, Cu, Se, V, Zn, Mg, CAT enzyme activity, TAS and GSH can be associated risk factors of CAD and should be considered together with CRP, LDL-C, total cholesterol and triglycerides monitoring for development of CAD. In this study, the results clearly indicate that CAD can be caused by oxidative stress and chronic inflammation.

Coronary artery risk factors (total cholesterol, CRP, triglycerides) were correlated with serum Ca, Cu and albumin levels. The results suggest that Cu, albumin and Ca levels may have a predictive value for healthy subjects. In the current study, the serum Zn level was found to be considered significantly lower in patients with CAD compared with the control subjects; moreover, it was found to be correlated with the SOD enzyme activity SOD-Zn (r=0.399; *P*=0.029). However, no significant correlations were found between GSH-Px, CAT, MDA, TOS, OSI, TSA, As, Be, Cd, Cr, Li, Pb, Sb, Sr, Ti, V, Na, BUN and UA parameters in CAD patients (BS).

## CONCLUSION

As a result of the analysis, it was observed that, in terms of changes in V/Zn ratio, MDA, TSA, LSA, TOS, OSI, Co, Cu, Se, V, Zn, Mg, CAT enzyme activity, TAS and GSH levels, the relationship between oxidative stress and inflammation was found in different groups of cardiac surgery patients who underwent cardiopulmonary bypass.

In the correlation analyses, we found a significant association between LDL-C-total cholesterol, triglycerides-Ca, SOD-Zn, CRP-albumin and total cholesterol-Cu. Our results showed that increased levels of LDL-C, CRP and triglycerides have also been found to be directly related to increased risk of CAD. It was concluded that the levels of LDL-C, total cholesterol, triglycerides and inflammatory markers such as CRP, significantly associated with parameters, as well as Cu, Ca and SOD enzyme activity, should be measured together for monitoring CAD. Our study also suggests that measurement of TSA and MDA might be an appropriate choice for biomarkers of CAD.

**Table:** 

Authors' roles & responsibilities	
DY	Substantial contributions to the conception or design of the work; or the acquisition, analysis, or interpretation of data for the work; drafting the work or revising it critically for important intellectual content; final approval of the version to be published
SE	Substantial contributions to the conception or design of the work; or the acquisition, analysis, or interpretation of data for the work; drafting the work or revising it critically for important intellectual content; final approval of the version to be published
SS	Substantial contributions to the conception or design of the work; or the acquisition, analysis, or interpretation of data for the work; drafting the work or revising it critically for important intellectual content; final approval of the version to be published
